# Supplementary material for: Diffusion probabilistic models enhance variational autoencoder for crystal structure generative modeling
Source: Sci Rep. 2024 Jan 13;14:1275. doi: 10.1038/s41598-024-51400-4 (PMC10787843; doi:10.1038/s41598-024-51400-4)
Supplement: Supplementary file 1 — Supplementary Information. [file 41598_2024_51400_MOESM1_ESM.pdf]

Supplementary Information: Diffusion probabilistic models enhance variational autoencoder for crystal structure generative modeling

Teerachote Pakornchote,[1] Natthaphon Choomphon-anomakhun,[1] Sorrjit Arrerut,[1] Chayanon Atthapak,[1,2] Sakarn Khamkaeo,[1,2] Thiparat Chotibut,[3] and Thiti Bovornratanaraks*[1,2]

[1] *Extreme Conditions Physics Research Laboratory and Center of Excellence in Physics of Energy Materials (CE:PEM), Department of Physics, Faculty of Science, Chulalongkorn University, Bangkok 10330, Thailand*
[2] *Thailand Center of Excellence in Physics , Ministry of Higher Education, Science, Research and Innovation, 328 Si Ayutthaya Road, Bangkok 10400, Thailand*
[3] *Chula Intelligent and Complex Systems, Department of Physics, Faculty of Science, Chulalongkorn University, Bangkok 10330, Thailand*

(Dated: November 23, 2023)

ENCODERS

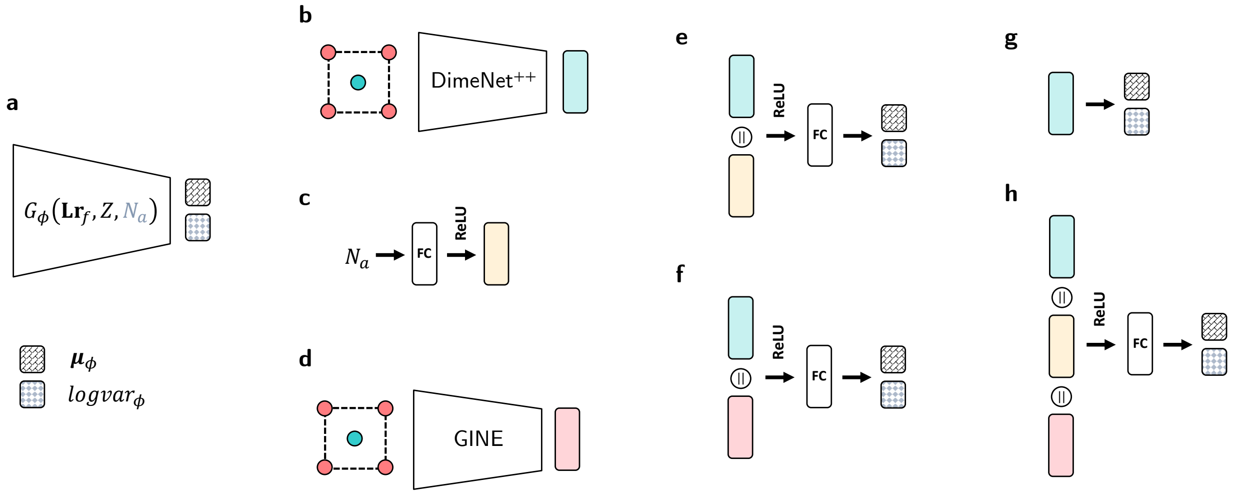

FIG. S1. (a) is the encoder for predicting $\boldsymbol{\mu}_\phi$ and $logvar_\phi$. (b) and (d) are DimeNet^{++} and GINE encoders, respectively, that take pristine crystal structures as inputs. (c) is the N_a encoder that takes the number of atoms N_a as an input. (e), (f), (g), and (h) are encoders of DP-CDVAE+N_a, DP-CDVAE+GINE, DP-CDVAE, and DP-CDVAE+N_a+GINE models, respectively. Blue, yellow, and pink boxes are the latent features from (b), (c), and (d), respectively, and FC boxes are fully connected layers.

MODEL EVALUATION

TABLE S1. Reconstruction performance using Eq. 6.

Models	Match rate (%) ↑			$\langle\delta_{\rm rms}\rangle$ ↓		
	Perov-5	Carbon-24	MP-20	Perov-5	Carbon-24	MP-20
DP-CDVAE	68.72	35.86	20.40	0.0177	0.2573	0.0715
DP-CDVAE+N_a	67.56	38.13	21.71	0.0222	0.2719	0.0774
DP-CDVAE+GINE	49.56	35.27	19.98	0.0822	0.2683	0.0620
DP-CDVAE+N_a+GINE	64.12	34.68	24.43	0.0250	0.3212	0.0697

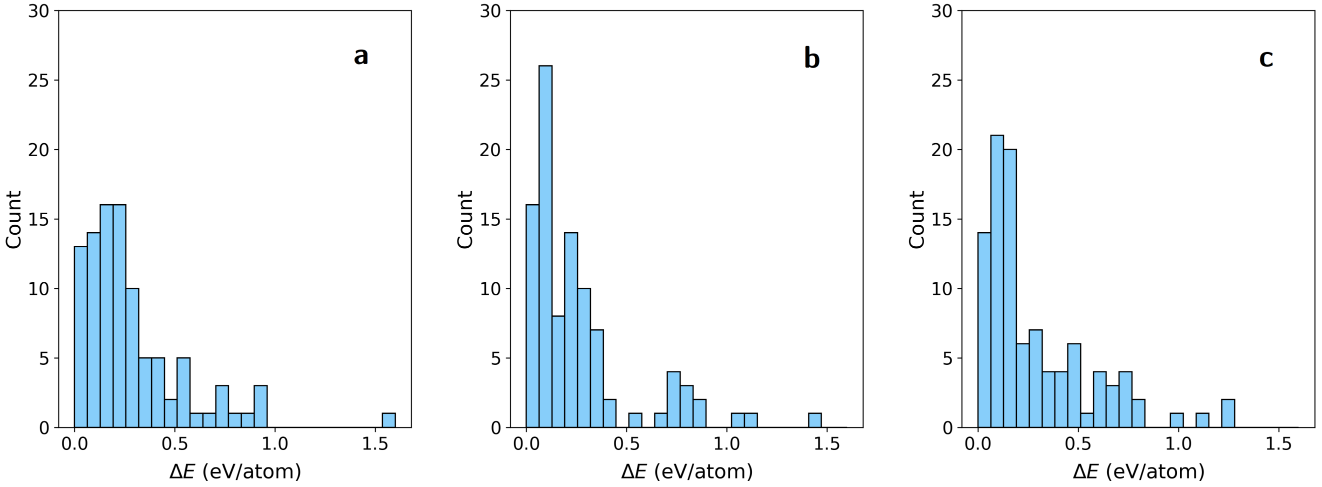

FIG. S2. Histograms of energy difference between generated and relaxed structures of (a) CDVAE, (b) CDVAE+Fourier, and (c) DP-CDVAE models. The modes (highest counting number) of CDVAE, CDVAE+Fourier, and DP-CDVAE models are 19.2 – 32.0, 68.0 – 128, and 64.0 – 128 eV/atom, respectively, where the bin size is 64.0 meV/atom.

FOURIER FEATURES

We appended the Fourier features to the node attributes for the input of the diffusion network. The Fourier features are the concatenation of $\sin(2^n\pi\mathbf{r}_t)$ and $\cos(2^n\pi\mathbf{r}_t)$ where $n \in \{n_{\min}, ..., n_{\max}\}$. For every DP-CDVAE model in the main text, $n_{\min} = 3$ and $n_{\max} = 8$.

LOSS FUNCTIONS

Similar to the original CDVAE, the total loss function to train the model consists of 5 sub-loss functions: the Kullback–Leibler divergence loss ($\mathcal{L}_{KLD}$) for training $\boldsymbol{\mu}_\phi$ and $logvar_\phi$, the lattice loss ($\mathcal{L}_{latt}$) for training lattice parameters, the composition loss ($\mathcal{L}_{comp}$) for training $\mathbf{A_z}$, the loss for training the number of atoms ($\mathcal{L}_{N_a}$), and the loss from the diffusion network ($\mathcal{L}_{diff}$) for training $\boldsymbol{\epsilon}_\theta$ and $\mathbf{A}_\theta$. In particular, the total loss is

$$\mathcal{L} = \mathcal{L}_{diff} + \lambda_1\mathcal{L}_{KLD} + \lambda_2\mathcal{L}_{latt} + \lambda_3\mathcal{L}_{comp} + \lambda_4\mathcal{L}_{N_a}, \quad \text{(S1)}$$

where λ_1, λ_2, λ_3, and λ_4 are tunable loss scaling factors, $\mathcal{L}_{latt}$ is computed from the mean square error of lattice parameters, and $\mathcal{L}_{comp}$ and $\mathcal{L}_{N_a}$ are cross-entropy losses.

---

[1] R. J. Needs and C. J. Pickard, Perspective: Role of structure prediction in materials discovery and design, APL Materials **4**, 053210 (2016).

[2] W. Kohn and L. J. Sham, Phys. Rev. **140**, A1133 (1965).

[3] A. R. Oganov and C. W. Glass, Crystal structure prediction using ab initio evolutionary techniques: Principles and applications, The Journal of Chemical Physics **124**, 244704 (2006).

[4] Y. Wang, J. Lv, L. Zhu, and Y. Ma, Crystal structure prediction via particle-swarm optimization, Phys. Rev. B **82**, 094116 (2010).

[5] A. R. Oganov, C. J. Pickard, Q. Zhu, and R. J. Needs, Structure prediction drives materials discovery, Nature Reviews Materials **4**, 331 (2019).

[6] J. C. Schön, K. Doll, and M. Jansen, Predicting solid compounds via global exploration of the energy landscape of solids on the ab initio level without recourse to experimental information, physica status solidi (b) **247**, 23 (2010).

[7] E. V. Podryabinkin, E. V. Tikhonov, A. V. Shapeev, and A. R. Oganov, Accelerating crystal structure prediction by machine-learning interatomic potentials with active learning, Phys. Rev. B **99**, 064114 (2019).

[8] C. J. Pickard and R. J. Needs, Ab initio random structure searching, Journal of Physics: Condensed Matter **23**, 053201 (2011).

[9] C. Shi, S. Luo, M. Xu, and J. Tang, Learning gradient fields for molecular conformation generation, in *Proceedings of the 38th International Conference on Machine Learning*, Proceedings of Machine Learning Research, Vol. 139, edited by M. Meila and T. Zhang (PMLR, 2021) pp. 9558–9568.

[10] T. Xie, X. Fu, O.-E. Ganea, R. Barzilay, and T. S. Jaakkola, Crystal diffusion variational autoencoder for periodic material generation, in *International Conference on Learning Representations* (2022).

[11] M. Xu, L. Yu, Y. Song, C. Shi, S. Ermon, and J. Tang, Geodiff: A geometric diffusion model for molecular conformation generation, in *International Conference on Learning Representations* (2022).

[12] J. Guan, W. W. Qian, X. Peng, Y. Su, J. Peng, and J. Ma, 3d equivariant diffusion for target-aware molecule generation and affinity prediction, in *The Eleventh International Conference on Learning Representations* (2023).

[13] Y. Song and S. Ermon, Generative modeling by estimating gradients of the data distribution, in *Advances in Neural Information Processing Systems*, Vol. 32, edited by H. Wallach, H. Larochelle, A. Beygelzimer, F. d'Alché-Buc, E. Fox, and R. Garnett (Curran Associates, Inc., 2019).

[14] Y. Song, J. Sohl-Dickstein, D. P. Kingma, A. Kumar, S. Ermon, and B. Poole, Score-based generative modeling through stochastic differential equations, in *International Conference on Learning Representations* (2021).

[15] D. P. Kingma and M. Welling, Auto-encoding variational bayes, in *International Conference on Learning Representations* (2014).

[16] J. Ho, A. Jain, and P. Abbeel, Denoising diffusion probabilistic models, in *Advances in Neural Information Processing Systems*, Vol. 33, edited by H. Larochelle, M. Ranzato, R. Hadsell, M. Balcan, and H. Lin (Curran Associates, Inc., 2020) pp. 6840–6851.

[17] R. Jiao, W. Huang, P. Lin, J. Han, P. Chen, Y. Lu, and Y. Liu, Crystal structure prediction by joint equivariant diffusion on lattices and fractional coordinates, in *Workshop on "Machine Learning for Materials" ICLR 2023* (2023).

[18] S. Kang and K. Cho, Conditional molecular design with deep generative models, Journal of Chemical Information and Modeling **59**, 43 (2019), pMID: 30016587.

[19] J. Lim, S. Ryu, J. W. Kim, and W. Y. Kim, Molecular generative model based on conditional variational autoencoder for de novo molecular design, Journal of Cheminformatics **10**, 31 (2018).

[20] Y. Song, L. Shen, L. Xing, and S. Ermon, Solving inverse problems in medical imaging with score-based generative models, in *International Conference on Learning Representations* (2022).

[21] M. R. Carbone, M. Topsakal, D. Lu, and S. Yoo, Machine-learning x-ray absorption spectra to quantitative accuracy, Phys. Rev. Lett. **124**, 156401 (2020).

[22] Z. Liang, M. R. Carbone, W. Chen, F. Meng, E. Stavitski, D. Lu, M. S. Hybertsen, and X. Qu, Decoding structure-spectrum relationships with physically organized latent spaces, Phys. Rev. Mater. **7**, 053802 (2023).

[23] A. Cui, K. Jiang, M. Jiang, L. Shang, L. Zhu, Z. Hu, G. Xu, and J. Chu, Decoding phases of matter by machine-learning raman spectroscopy, Phys. Rev. Appl. **12**, 054049 (2019).

[24] A. Okhotin, D. Molchanov, V. Arkhipkin, G. Bartosh, A. Alanov, and D. Vetrov, Star-shaped denoising diffusion probabilistic models (2023), arXiv:2302.05259 [stat.ML].

[25] M. M. Bronstein, J. Bruna, T. Cohen, and P. Velickovic, Geometric deep learning: Grids, groups, graphs, geodesics, and gauges, CoRR **abs/2104.13478** (2021), 2104.13478.

[26] T. S. Cohen, M. Geiger, J. Köhler, and M. Welling, Spherical CNNs, in *International Conference on Learning Representations* (2018).

[27] N. Thomas, T. Smidt, S. Kearnes, L. Yang, L. Li, K. Kohlhoff, and P. Riley, Tensor field networks: Rotation- and translation-equivariant neural networks for 3d point clouds (2018).

[28] Z. Ren, S. I. P. Tian, J. Noh, F. Oviedo, G. Xing, J. Li, Q. Liang, R. Zhu, A. G. Aberle, S. Sun, X. Wang, Y. Liu, Q. Li, S. Jayavelu, K. Hippalgaonkar, Y. Jung, and T. Buonassisi, An invertible crystallographic representation for general inverse design of inorganic crystals with targeted properties, Matter **5**, 314 (2022).

[29] J. Gasteiger, J. Groß, and S. Günnemann, Directional message passing for molecular graphs, in *International Conference on Learning Representations* (2020).

[30] J. Gasteiger, S. Giri, J. T. Margraf, and S. Günnemann, Fast and uncertainty-aware directional message passing for non-equilibrium molecules (2022), arXiv:2011.14115 [cs.LG].

[31] K. Xu, W. Hu, J. Leskovec, and S. Jegelka, How powerful are graph neural networks?, in *International Conference on Learning Representations* (2019).

[32] W. Hu*, B. Liu*, J. Gomes, M. Zitnik, P. Liang, V. Pande, and J. Leskovec, Strategies for pre-training graph neural networks, in *International Conference on Learning Representations* (2020).

[33] K. Schütt, P.-J. Kindermans, H. E. Sauceda Felix, S. Chmiela, A. Tkatchenko, and K.-R. Müller, Schnet: A continuous-filter convolutional neural network for modeling quantum interactions, in *Advances in Neural Information Processing Systems*, Vol. 30, edited by I. Guyon, U. V. Luxburg, S. Bengio, H. Wallach, R. Fergus, S. Vishwanathan, and R. Garnett (Curran Associates, Inc., 2017).

[34] J. Klicpera, F. Becker, and S. Günnemann, Gemnet: Universal directional graph neural networks for molecules, in *Advances in Neural Information Processing Systems*, edited by A. Beygelzimer, Y. Dauphin, P. Liang, and J. W. Vaughan (2021).

[35] D. P. Kingma, T. Salimans, B. Poole, and J. Ho, On density estimation with diffusion models, in *Advances in Neural Information Processing Systems*, edited by A. Beygelzimer, Y. Dauphin, P. Liang, and J. W. Vaughan (2021).

[36] R. W. Grosse-Kunstleve, N. K. Sauter, and P. D. Adams, Numerically stable algorithms for the computation of reduced unit cells, Acta Crystallographica Section A **60**, 1 (2004).

[37] I. E. Castelli, D. D. Landis, K. S. Thygesen, S. Dahl, I. Chorkendorff, T. F. Jaramillo, and K. W. Jacobsen, New cubic perovskites for one-and two-photon water splitting using the computational materials repository, Energy & Environmental Science **5**, 9034 (2012).

[38] I. E. Castelli, T. Olsen, S. Datta, D. D. Landis, S. Dahl, K. S. Thygesen, and K. W. Jacobsen, Computational screening of perovskite metal oxides for optimal solar light capture, Energy & Environmental Science **5**, 5814 (2012).

[39] A. Jain, S. P. Ong, G. Hautier, W. Chen, W. D. Richards, S. Dacek, S. Cholia, D. Gunter, D. Skinner, G. Ceder, *et al.*, Commentary: The materials project: A materials genome approach to accelerating materials innovation, APL materials **1**, 011002 (2013).

[40] C. J. Pickard,.

[41] C. K. Joshi, C. Bodnar, S. V. Mathis, T. Cohen, and P. Lio, On the expressive power of geometric graph neural networks (2023).

[42] Y. Wu, P. Lazic, G. Hautier, K. Persson, and G. Ceder, First principles high throughput screening of oxynitrides for water-splitting photocatalysts, Energy Environ. Sci. **6**, 157 (2013).

[43] T. Ishikawa and T. Miyake, Evolutionary construction of a formation-energy convex hull: Practical scheme and application to a carbon-hydrogen binary system, Phys. Rev. B **101**, 214106 (2020).

[44] A. Ektarawong, E. Johansson, T. Pakornchote, T. Bovornratanaraks, and B. Alling, Boron vacancy-driven thermodynamic stabilization and improved mechanical properties of alb2-type tantalum diborides as revealed by first-principles calculations, Journal of Physics: Materials **6**, 025002 (2023).

[45] B. Gruber, The relationship between reduced cells in a general Bravais lattice, Acta Crystallographica Section A **29**, 433 (1973).

[46] I. Křivý and B. Gruber, A unified algorithm for determining the reduced (Niggli) cell, Acta Crystallographica Section A **32**, 297 (1976).

[47] A. Q. Nichol and P. Dhariwal, Improved denoising diffusion probabilistic models, in *Proceedings of the 38th International Conference on Machine Learning*, Proceedings of Machine Learning Research, Vol. 139, edited by M. Meila and T. Zhang (PMLR, 2021) pp. 8162–8171.

[48] P. E. Blöchl, Phys. Rev. B **50**, 17953 (1994).

[49] G. Kresse and J. Furthmüller, Computat. Mater. Sci. **6**, 15 (1996).

[50] G. Kresse and J. Furthmüller, Phys. Rev. B **54**, 11169 (1996).
[51] J. P. Perdew, K. Burke, and M. Ernzerhof, Phys. Rev. Lett. **77**, 3865 (1996).
[52] H. J. Monkhorst and J. D. Pack, Phys. Rev. B **13**, 5188 (1976).
[53] J. D. Pack and H. J. Monkhorst, Phys. Rev. B **16**, 1748 (1977).
